# Efficacy of Olyset^®^ Duo, a permethrin and pyriproxyfen mixture net against wild pyrethroid-resistant *Anopheles gambiae s.s.* from Côte d’Ivoire: an experimental hut trial

**DOI:** 10.1051/parasite/2015028

**Published:** 2015-10-21

**Authors:** Alphonsine A. Koffi, Ludovic P. Ahoua Alou, Armel Djenontin, Jean-Paul K. Kabran, Youssouf Dosso, Aboubacar Kone, Nicolas Moiroux, Cedric Pennetier

**Affiliations:** 1 Institut Pierre Richet (IPR), Institut National de Santé Publique (INSP) Bouaké Côte d’Ivoire; 2 Institut de Recherche pour le Développement (IRD), Maladies Infectieuses et Vecteurs, Ecologie, Génétique, Evolution et Contrôle (MIVEGEC), UM-CNRS 5290 IRD 224 Cotonou Benin; 3 Faculté des Sciences et Techniques-Université d’Abomey-Calavi Cotonou Benin; 4 Centre de Recherche Entomologique de Cotonou (CREC) Cotonou Benin; 5 IRD MIVEGEC, UM-CNRS 5290 IRD 224 Montpellier France

**Keywords:** *Anopheles gambiae*, pyrethroid insecticide, resistance, bed net, insect growth regulator

## Abstract

Pyrethroid resistance in malaria vectors has spread across sub-Saharan Africa. Alternative tools and molecules are urgently needed for effective vector control. One of the most promising strategies to prevent or delay the development of resistance is to use at least two molecules having unrelated modes of action in combination in the same bed net. We evaluated in experimental huts in Côte d’Ivoire, a new polyethylene long-lasting insecticidal net (LN) product, Olyset^®^ Duo, incorporating permethrin (PER) and pyriproxyfen (PPF), an insect growth regulator (IGR). PPF alone or in combination with permethrin had a significant impact on fertility (7–12% reduction relative to control) and no effect on fecundity of wild multi-resistant *An. gambiae s.s.* These results triggered crucial research questions on the behaviour of targeted mosquitoes around the LN. To maximize the sterilizing effect of PPF in the combination, there would be a need for a trade-off between the necessary contact time of the insect with PPF and the surface content of the pyrethroid insecticide that is bioavailable and induces excito-repellency.

## Abstract (fr)

La résistance aux pyréthrinoïdes est répandue chez les vecteurs du paludisme en Afrique sub-saharienne. Une des stratégies les plus prometteuses pour prévenir ou retarder l’évolution de la résistance est d’utiliser au moins deux molécules ayant des modes d’action indépendants en combinaison dans la même moustiquaire. Nous avons évalué en cases expérimentales en Côte d’Ivoire, une nouvelle moustiquaire à longue durée d’action (MILD) en polyéthylène, Olyset^®^ Duo, associant la perméthrine (un insecticide pyréthrinoïde) et le pyriproxyfen (un inhibiteur de croissance). Le pyriproxyfen seul ou combiné à la perméthrine a induit une réduction significative de la fertilité (7-12 % de réduction par rapport au contrôle) mais n’a eu aucun effet sur la fécondité des femelles sauvages multi-résistantes. Ces résultats ont soulevé des questions cruciales de recherche sur le comportement des moustiques cibles face aux MILD. Pour maximiser l’effet stérilisant du PPF dans la combinaison, il y aurait un besoin de compromis entre le temps de contact nécessaire de l’insecte avec le PPF et le contenu de surface de l’insecticide pyréthrinoïde qui est biodisponible et induit l’excito-répulsion.

## Introduction

Insecticide-treated bed nets are currently the cornerstone of malaria transmission prevention in Africa. To date, only pyrethroids are used for bed net treatment because they meet the necessary safety criteria [28]. For almost a decade, mass distribution of long-lasting insecticidal net (LNs) has been scaled-up across sub-Saharan Africa with support from the Global Fund to Fight AIDS, Tuberculosis and Malaria [28]. Unfortunately, there are increasing reports of pyrethroid resistance associated with reduced vector mortality and drastic loss of personal protection that pyrethroid-treated nets confer to humans [1, 3, 15, 18]. The operational impact of metabolic-based resistance and target site mutations is still largely overlooked and even controversial [22].

Pyrethroid-resistance is now widespread among malaria vector populations [20, 21], and its rapid and significant increase could undermine malaria control [26]. Faced with this threat, alternative tools and molecules are urgently needed for effective vector control. One of the most promising strategies to prevent or delay the development of resistance is to use at least two molecules having unrelated modes of action in combination in the same bed net. The rationale is that mosquitoes resistant to one insecticide should theoretically be killed by the other component. Combination nets are likely to be more effective than standard nets in areas with resistant malaria vectors [2]. Therefore, the use of mixtures is considered as an important strategy in the Global Plan for Insecticide Resistance Management (GPIRM) in malaria vectors [26]. During the last few years, new LNs claimed as resistance breaking products have emerged, some of them impregnated with a combination of a pyrethroid with the synergist pyperonyl butoxide (PBO) and have shown promising results [3, 14, 19, 23, 24].

Sumitomo Chemical recently developed a new LN product, so-called Olyset^®^ Duo, made of polyethylene incorporating permethrin (PER) and pyriproxyfen (PPF), an insect growth regulator (IGR). PPF is a juvenile hormone analogue (JHA) which inhibits metamorphosis and embryogenesis in several mosquitoes [5] and is already widely used as a larvicide [4]. The idea is that mosquitoes surviving contact with the net, because they are resistant to permethrin, will be sterilized by the PPF after contact with the mixture LN. Nets treated with PPF showed good performance in the laboratory by sterilizing and shortening the longevity of *Anopheles gambiae* females after tarsal contact [17]. In a twin paper, we also reported insecticidal and sterilizing performances through release-recapture experiments run in experimental huts in Benin against both susceptible and resistant laboratory strains [6]. The resistant strain used in the later study was homozygous resistant for the *kdr* L1014F mutation. Promising results in terms of sterilizing effect have been observed among surviving resistant females. It was then necessary to investigate the performances of such LNs against wild free-flying multi-resistant [9, 10] *An. gambiae s.s.* in experimental huts.

The analysis of fecundity and fertility considered pyrethroid-resistant *An. gambiae* females that blood-fed and survived contact with permethrin on the net surface. In order to obtain enough surviving blood-fed females and fulfil our objectives, a variable consisting of the number of 4 cm × 4 cm holes was introduced in the study: LNs containing 6, 30 or 150 holes were used in the release-recapture study [6]. The results of the study indicated that PPF alone drastically reduced fecundity by 98% and fertility by 93% in laboratory-reared *An. gambiae* having *kdr* mutation as a resistance mechanism. More importantly, we showed that a number of holes as low as 6 or 30 in Olyset^®^ Duo drastically reduced the mean number of eggs of the pyrethroid-resistant surviving blood-fed females, while the same LN with larger number of holes (150) did not. It is suggested that the number of holes combined with the irritant effect of permethrin limited the contact between the females and the treated surface, reducing mosquito uptake of sufficient PPF.

The challenge of the Phase II trial was to collect enough surviving blood-fed females in the hut with the Olyset^®^ Net and Olyset^®^ Duo. The objective was to have a sufficient number of surviving blood-fed females without compromising the actual contact time mosquitoes spent probing through the PPF-treated net. This was done to avoid underestimating the LN protective effect. For this reason, the configuration with 30 holes was chosen to be tested in experimental hut in M’Bé, central Côte d’Ivoire against wild free-flying *Anopheles gambiae* mosquitoes resistant to most current public health insecticides [9]. Its insecticidal and protective efficacy was expressed in terms of deterrence, blood‐feeding inhibition, induced exophily and induced mortality. Its sterilizing performance was expressed in terms of reduction of fecundity and fertility of the females that survived treatment exposure.

## Materials and methods

### Experimental hut trial

#### Study area

The Phase II trial was carried out in experimental huts located in M’bé (Côte d’Ivoire). The M’bé valley is a huge rice‐growing area situated 30 km to the North of Bouaké (5.209963 W and 7. 970241 N) in Central Côte d’Ivoire. Bouaké is a transitional zone characterized by wet savannah. This region has one rainy season (April to October) and an average annual rainfall of 1200 mm and an average annual temperature of 25.8 °C. The mosquito population in the area is composed of *An. gambiae s.s.*, *An. funestus*, *Culex* sp. and *Mansonia* sp. Both *An. coluzzii* and *An. gambiae s.s.* of *An. gambiae s.s.* co‐exist in sympatry but *An. coluzzii* is largely dominant (almost 99% of the population) [9]. The *An. gambiae s.s.* population is resistant to organochlorides, pyrethroids and carbamates with an allelic frequency of the L104F *kdr* mutation around 30% and the presence of metabolic resistance mechanisms [9].

#### Design of huts

The huts were made from concrete bricks, with a corrugated iron roof, a ceiling of thick polyethylene sheeting and a concrete base surrounded by a water‐filled channel to prevent entry of ants [25]. Mosquito access was via four window slits constructed from pieces of metal, fixed at an angle to create a funnel with a 1 cm wide gap. Mosquitoes fly upward to enter through the gap and downwards to exit, this precludes or greatly limits exodus though the aperture enabling the majority of entering mosquitoes to be accounted for. A single veranda trap made of polyethylene sheeting and screening mesh measuring 2 m long, 1.5 m wide and 1.5 m high, projects from the back wall of each hut. Movement of mosquitoes between hut and veranda is unimpeded during the night.

#### Study design

The following treatment arms were tested:

- Olyset^®^ Net, a permethrin 2% (w/w) incorporated into polyethylene net.
- Pyriproxyfen (PPF) 1% (w/w) incorporated into polyethylene net.
- Olyset^®^ Duo, a permethrin 2% (w/w) + pyriproxyfen 1% (w/w) incorporated into polyethylene net,
- Untreated polyethylene net.

Before evaluation in experimental huts, the nets (including control) were deliberately holed with 30 holes each of 16 cm^2^ (4 cm × 4 cm) to simulate a worn net. Nets were washed three times according to the WHO Pesticide Evaluation Scheme (WHOPES) standard washing methods 1 week before the trial [27].

Adult volunteer sleepers spent six nights per week under the mosquito nets in the experimental huts. They were rotated randomly among huts each night of the study. They entered the hut at dusk and slept until dawn. Once every week, the huts were thoroughly cleaned and aired to avoid contamination.

Each morning, dead and living mosquitoes were collected from the floor of the huts, the veranda traps and inside the nets. Mosquitoes were morphologically identified to species using taxonomic keys and were scored by location as dead or live and as fed or unfed. Live mosquitoes were placed in small netted plastic cups and supplied with 10% honey solution to assess delayed mortality after 24 h.

The primary outcomes were the ones usually measured in experimental hut trial:

- deterrence by the treatments (i.e. the reduction in the number of mosquitoes in huts with LNs relative to the control untreated net);
- induced exophily (IE) (i.e. the proportion of mosquitoes found in exit traps in huts with LNs relative to the total collected in hut with untreated net);
- blood‐feeding inhibition (BFI) (i.e. the reduction in blood-feeding of mosquitoes in huts with LNs relative to the hut with control untreated net);
- immediate and delayed mortality (i.e. the proportion of dead mosquitoes at the time of collection in the morning and after 24 h holding, corrected from untreated control mortality).

#### Reporting of adverse events

The volunteers were asked to report any adverse events associated with use of nets and a provision for medical care was made.

### Fecundity and fertility assessment

Alive blood-fed females collected from all compartments of the huts were counted. All the blood-fed females per hut were put by batch of up to 20 females in cardboard cups (450 mL). We previously filled the bottom of the cardboard cups with 1 cm high layer of wetted cotton, covered with a filter paper disc to allow females to lay their eggs. Females in cardboard cups were maintained with honey solution at 28 °C and 80% RH for 5 days before checking mortality. A picture of each filter paper was taken to count the eggs using egg counter software [13]. All eggs from the control and treated batches were immersed in water. After 6 days, the number of larvae was checked to determine the hatching rates. Because pyriproxyfen acts by sterilizing the adult female mosquito, the impact of the treatments on the reproduction of surviving blood-fed mosquitoes was investigated by detecting whether there was a reduction in fecundity (number of eggs per female) and fertility (proportion of laid eggs hatching) of these mosquitoes compared to the control.

#### LN bioassays

Standard WHO cone bioassays were used to determine bio-efficacy of LNs against a susceptible laboratory-reared *Anopheles gambiae* Kisumu strain [27]. Four nets (one per treatment arm) were bioassayed at time 0 (i.e. the day before the first washing). Bioassays were carried out for a second time after washings and then for a third time at the end of the field trial with nets used in the huts. For each net, five cones were placed on the five sections of the net (roof and four sides). Ten unfed Kisumu females, 2–3 days old, were introduced per cone and exposed for 3 min to each. Knockdown (KD) was checked 60 min after exposure and mortality was recorded 24 h after exposure. Mosquitoes exposed to untreated nets were used as controls and Abbott’s adjustment applied if mortality was >5% for the controls.

### Statistical analysis

The software “R” was used for the statistical analyses [20]. The proportion of mosquitoes that exited early, successfully blood-fed or died was analysed using a logistic regression model. The “brglm” function from the brglm package was used for the analysis [11]. It enables fitting of binomial-response regression models using the bias-reduction method developed in Firth [7]. These procedures return estimates with improved frequentist properties (bias, mean squared error) that are always finite even in cases where the maximum likelihood estimates are infinite (data separation). The number of collected mosquitoes entering the huts was analysed using negative binomial regression. Mortality and KD rates from WHO cone bioassays were compared between each net using the χ^2^ test. For statistical testing the level of significance was set at 5%.

### Ethical considerations

Ethical approval was obtained from the Ministry of Health and Fight against AIDS in Côte d’Ivoire through the National Ethics Committee. Adult volunteers were recruited among the inhabitants of the villages close to the site. Volunteer sleepers were recruited after obtaining informed written consent. Medical supervision was provided throughout the trial and for an additional one month after the end of the study by a qualified medical doctor. Confirmed malaria cases were treated with “artesunate + amodiaquine”, according to national policies, and all the volunteer sleepers were vaccinated against yellow fever after enrolment.

## Results

### Bio‐efficacy of the treated nets (WHO cone bioassays)

Table 1 shows the bio-efficacy of each comparison arm in terms of KD effect and mortality before washing, after three washes and after the field trial. Before washing, KD rates recorded with the *An. gambiae s.s.* susceptible Kisumu strain were 100% for both Olyset^®^ Net and Olyset^®^ Duo. Olyset^®^ Duo caused 100% mortality whereas Olyset^®^ Net induced significantly lower mortality (22% mortality; *p* < 0.0001). The control and PPF nets did not induce any KD effect or mortality.

**Table 1.** Knockdown (KD) rate at 60 min and mortality rate of *An. gambiae* Kisumu strain after 3 min exposure to treated nets following WHO standard procedures (WHO 2013) run before washes (28/09/2012), after the three washes (10/10/2012), and after the trial (13/11/2012).

Treatment	Before any washing			After three washes and prior to field trial			After washing and field trial		
	*N*	% KD (60 min)	% Mort. (24 h)	*N*	% KD (60 min)	% Mort. (24 h)	*N*	% KD (60 min)	% Mort. (24 h)
Control (Untreated net)	49	0^a,1^	0^a,1^	50	0^a,1^	0^a,1^	55	2^a,1^	2^a,1^
Pyriproxyfen treated LN	46	0^a,1^	0^a,1^	54	0^a,1^	0^a,1^	50	0^a,1^	0^a,1^
Olyset Net	50	100^b,3^	22^b,2^	50	44^b,2^	0^a,1^	61	62^b,2^	13^b,2^
Olyset Duo	48	100^b,1^	100^c,3^	50	100^c,1^	66^b,1^	49	96^c,1^	78^c,2^

*N*: Number of females tested, KD: Knockdown observed 60 min after exposure; Mort.: Mortality recorded 24 h post-exposure. Values in the same column sharing the same letter superscript do not differ significantly (*p* > 0.05) according to χ^2^ tests. For KD and mortality, values in the same line sharing the same superscript number do not differ significantly (*p* > 0.05) according to χ^2^ tests.

After washing and prior to the field trial, the control and PPF nets did not induce any KD effect or mortality. With Olyset^®^ Net, KD and mortality decreased significantly (*p* < 0.05) from before washing. With Olyset^®^ Duo, only mortality decreased significantly (*p* < 0.001) after washing.

After the field trial, KD rate increased significantly (*p* < 0.05) for Olyset^®^ Net, but remained stable for Olyset^®^ Duo (*p* > 0.05). Mortality increased significantly (*p* < 0.05) for Olyset^®^ Duo and Olyset^®^ Net (*p* < 0.05) from the level observed just after washing. The PPF-treated nets induced neither KD effect nor mortality (Table 1). Overall, Olyset^®^ Duo outperformed the other treatments before and after the field trial.

### Insecticidal efficacy of treatments during the field trial

Mosquito collection in experimental huts was carried out over 24 nights between October 8 and November 3, 2012. In total, 5056 *An. gambiae s.l.* and 3171 other mosquitoes were caught. Of the other mosquitoes caught, 96.6% were *Mansonia* spp., 1.4% were *Culex* spp., 0.4% were *Aedes* spp. and 1.8% were *Anopheles* species other than *An. gambiae s.s.* The results in terms of deterrence, induced exophily, blood-feeding inhibition and induced mortality are summarized in Table 2.

**Table 2.** Summary results of the experimental hut trial against wild free-flying *Anopheles gambiae s.l.* resistant to insecticides and other Culicidae

Treatments	Total collected	Deterrence (%)	Exophily			Blood-feeding			Mortality		
			*N* in VE	% caught in VE	IE (%)	*N* in BF	% BF	BFI (%)	*N* Dead	% mortality	% corrected mortality
	*An. gambiae s.s.*										
Control	1399^a^	–	638	45.6^a^	–	482	34.5^a^	–	110	7.9^a^	–
Pyriproxyfen net	1024^a^	NS	468	45.7^a^	NS	360	35.2^a^	NS	128	12.5^b^	5.0
Olyset Net	1431^a^	NS	936	65.4	43.42	744	52.0	−50.9	125	8.7^a^	NS
Olyset Duo	1202^a^	NS	815	67.8	48.68	453	37.7^a^	NS	177	14.7^b^	7.5
											
	Other Culicidae										
Control	818^a^	–	474	57.9	–	160	19.6	–	101	12.4^a^	–
Pyriproxyfen net	1339	−63.69	612	45.7^a^	−21.1	377	28.2	−43.9	182	13.6^a^	NS
Olyset Net	410	49.88	181	44.2^a^	−23.8	9	2.2^a^	88.8	277	67.6	63.0
Olyset Duo	604^a^	NS	227	37.6	−35.1	7	1.2^a^	94.1	505	83.6	81.0

VE: Veranda; IE: Induced exophily; BF: Blood-fed; BFI: Blood-feeding inhibition; NS: Not Significant, in both *An. gambiae s.s.* and other Culicidae categories, outcome measure values sharing the same superscript letter do not differ significantly (*p* > 0.05) according to negative binomial regression (total collected) or logistic regressions (BF and mortality).

#### *Anopheles gambiae s.s.*

During the trial, 1399 *An. gambiae s.l.* (i.e. a mean number of 58.3 per night) were caught in the control hut. We did not observe any deterrent effect by any treatment compared to the control (Table 2; *p* > 0.05).

Exophily for both Olyset^®^ Net and Olyset^®^ Duo was significantly higher (65.4% and 67.8%, respectively) than with the untreated net (45.6%; *p* < 0.001) whereas PPF-treated net (45.7%; *p* < 0.001). The induced exophily (IE) by the Olyset^®^ Duo (48.7%) was significantly higher than that by the Olyset^®^ Net treatment (43.4%; *p* < 0.01).

Under the untreated control net, 482 *An. gambiae s.l.* were blood-fed; this corresponds to 20.1 bites per person per night. Blood-feeding rates of *An. gambiae* were not significantly reduced by any of the treatments, relative to control (Table 2). Olyset^®^ Net produced a mortality rate of *An. gambiae s.l.* similar to that of the untreated net (7.86% vs. 8.74%; *p* > 0.05). The huts with PPF-treated net and Olyset^®^ Duo recorded significantly higher mortality of *An. gambiae s.l.* (12.5% and 14.7%, respectively) than the untreated net (*p* < 0.001). Mortality recorded with Olyset^®^ Duo was significantly higher than that with the Olyset^®^ Net (*p* < 0.01).

#### Other Culicidae

The mean number of Culicidae other than *An. gambiae s.s.* collected in the untreated hut was 34 per night. The only significant deterrence of these Culicidae entry rates to huts was with Olyset^®^ Net (49.9%; *p* < 0.001) (Table 2). None of the treatments repelled greater number of mosquitoes to the veranda than did the control untreated net. The proportion of other Culicidae blood-feeding under the untreated net was relatively low (19.6%). Nevertheless, significantly fewer mosquitoes blood-fed under the Olyset^®^ Net (2.2%) and Olyset^®^ Duo (1.2%), leading to BFI rates of 88.8% and 94.1%, respectively. The PPF-treated net by itself procured no protection against bites of these mosquitoes. Mortality rates of other Culicidae were 67.6% with Olyset^®^ Net and 83.6% with Olyset^®^ Duo, consistently higher than for *An. gambiae s.l*. (8.7% and 14.73%, respectively). The PPF-treated net killed negligible proportions of Culicidae (13.59%), similar to control (12.35%).

#### Adverse effects

No complaint or adverse effects (symptoms or disorders) in any treatment arm were reported by the sleepers during the experimental trial.

### Fecundity and fertility of surviving blood-fed females

During the study of fecundity and fertility, we scored 15,802 eggs laid by 1886 wild surviving blood-fed *An. gambiae* females and 8407 larvae. The results of fecundity and fertility are summarized in Table 3.

**Table 3.** Summary results of fecundity and fertility among the natural population of *An. gambiae s.s.* exposed to the different treatment arms

Treatment	Total collected	Survival		Fecundity		Fertility	
		*N* surviving blood-fed female	% surviving blood-fed	*N* eggs laid	*N* eggs/female	*N* larvae	% hatching
Control	1399	454	32^a^	2755	6^a^	1516	55^b^
Pyriproxyfen net	1024	332	32^a^	1729	5^a^	877	51^a^
Olyset^®^ Net	1431	692	48^b^	7540	11^a^	4186	56^b^
Olyset^®^ Duo	1202	408	34^a^	3778	9^a^	1828	48^a^

*N*: number; For each parameter, values in columns sharing the same superscript letter are not significantly different (*p* > 0.05) according to Negative Binomial regression (fecundity) or logistic regressions (survival and fertility).

The mean number of eggs/female ranged from 5 to 11 for all LN treatments, with no evidence that the LNs reduced fecundity of the wild pyrethroid-resistant *An. gambiae s.l.*, relative to fecundity of females in the control hut (*p* > 0.05).

The hatching rates were 55% from the hut with the untreated net, 51% in the PPF-treated net hut, 48% in the Olyset^®^ Duo and 56% in hut with the Olyset^®^ Net. The impact for the PPF and Olyset Duo was significant (7–12% reduction in fertility; *p* < 0.01).

## Discussion

We studied the insecticidal and sterilizing effect of a new LN impregnated with a mixture of permethrin and PPF against wild pyrethroid-resistant *An. gambiae s.s.* in Côte d’Ivoire. Bioassay on Olyset^®^ Duo showed greater insecticidal efficacy than Olyset^®^ Net and PPF-treated net against the standard susceptible *An. gambiae s.s.* Kisumu strain, reaching the WHOPES criteria (>95% KD or >80% mortality) after three washes and after the field trial. The fact that Olyset^®^ Duo and Olyset^®^ Net showed greater insecticidal efficacy after the field trial might be due to their different regeneration times and bleed rates [16]. The apparently poor performance of Olyset^®^ Net suggests that, in these conditions, this net required more time for full regeneration after the washing process. Gimnig et al. [8] showed that after washing, this net must be heated to 60 °C to restore bioefficacy, but a previous study [25] indicated possible full regeneration within 2 weeks at 30 °C and 80% RH. Nevertheless because the incorporation technology is different between LNs impregnated with one chemical (permethrin) and LNs impregnated with two chemicals (permethrin + PPF), the comparison will always suffer a bias. The results of such comparisons about KD and mortality dynamics must be interpreted carefully.

The Phase II trial was run in an area with strong resistance to insecticides and particularly permethrin where *An. gambiae s.l.* vectors bear both *kdr-w* and metabolic resistance mechanisms [9]. The results found in this study confirmed that the high resistance reported in this area had a strong impact on the efficacy of Olyset^®^ Net both in terms of personal protection through blood-feeding inhibition and mortality of *An. gambiae s.s.* While the Olyset^®^ Net was highly efficacious against other Culicidae mosquitoes, it performed poorly against wild multi‐resistant *An. gambiae s.s*., highlighting the crucial need for alternative tools for malaria vector control in areas of pyrethroid resistance. Emphasis is being put on use of the LN Olyset^®^ Duo that combines two molecules having unrelated modes of action for improved impact and potential management of permethrin resistance.

Olyset^®^ Duo induced significantly higher BFI and mortality than Olyset^®^ Net in M’Bé. This result was confirmed by the higher mortality rates in bioassays and release‐recapture trial with Olyset^®^ Duo than Olyset^®^ Net [6] and was similar to that found by Ngufor et al. [16]. The PPF did not show any insecticidal activity when used alone both in bioassays and release‐recapture, but it induced mortality (5% corrected from the control) against the multi‐resistant *An. gambiae s.s.* population of M’Bé. Ohashi et al. [17] have already shown that exposure to pyriproxyfen‐treated netting shortened the longevity of *An. gambiae s.s.* It might be interesting to investigate the interaction between permethrin and PPF to assess any relative contribution of PPF to the insecticidal effect of Olyset^®^ Duo. Olyset^®^ Duo having a higher permethrin bleed rate than Olyset Net – (J. Lucas, pers. comm.) may be a confounding factor that may render difficult the study of the interaction between permethrin and PPF.

As expected, there were a large number of blood-fed females surviving all treatments. Nevertheless, the egg laying rates were almost 10-fold lower in the control than during the release‐recapture trial [6]. Indeed, wild *An. gambiae s.s.* females had difficultly laid eggs under laboratory conditions compared to well-established *An. gambiae s.s.* strains in insectary. In this previous experiment [6], PPF alone or Olyset Duo containing 30 holes each drastically impacted fecundity of laboratory-reared *An. gambiae s.s.* having solely *kdr* as a resistance mechanism. In the current trial, the same LNs with the same amount of holes did not impact fecundity of the wild pyrethroid-resistant *An. gambiae s.s.* that in addition to *kdr* have metabolic resistance. Whether these additional metabolic resistances alone or in association with *kdr* impact negatively PPF has yet to be investigated.

The PPF activity or the Olyset^®^ Duo effects on the hatching rate (7–12% reduction) indicated that wild mosquitoes had some contact with the PPF active ingredient. Nevertheless, it is impossible to quantify this contact duration and the active ingredient quantity picked up by wild mosquitoes. We could hypothesize that the active ingredient quantity was enough to impact fertility but not fecundity.

It would be extremely interesting to investigate in laboratory conditions the relationship between the forced tarsal contact time with a PPF‐treated net and the impact on fecundity and fertility. These baseline data might allow us to better elucidate these observations in the field.

The behaviour of wild *An. gambiae s.s.* is different than those of *An. gambiae s.s.* strains reared in insectary [12]. Such behavioural differences might explain the difference in effect during the release-recapture experiment [6] and the current trial. Moreover using an IGR, such as PPF, on bed net is new; it is therefore important to update the evaluation criteria in order to allow fair evaluation of such new LN. To reach this goal, we suggest investigating the dynamics of the PPF activity in relation to the actual tarsal contact time mosquitoes spent on the net. Behavioural studies are also crucial to better develop this new strategy, especially the behaviour of aggressive *An. gambiae* mosquitoes in the presence of a torn net.

## Conclusion

In previous experiments [6], PPF alone or Olyset Duo containing 30 holes each drastically impacted fecundity and fertility in laboratory-reared *An. gambiae* having a *kdr* resistance mechanism. The current trial provided evidence that the same LNs with same amount of holes in them significantly impacted on fertility but did not impact on fecundity of the wild pyrethroid resistant *An. gambiae* that, in addition to *kdr*, have metabolic resistance. More trials of this kind should be conducted in other areas with other type of pyrethroid resistance for comparison. Moreover the difficulties experienced to correctly investigate the IGR performances highlighted the urgent need to deeply investigate both IGR intrinsic activity and mosquito behaviour in order to better understand the results observed in natural conditions. Whether these additional metabolic resistances alone or in association with *kdr* impact negatively PPF efficacy has yet to be investigated.

## Conflict of interest

This work was supported financially by Sumitomo Chemical. Funders participated in the study design and the decision to publish, but they have no role in data collection, analysis and preparation of the manuscript. The authors declare that they have no competing interests.
